# Healthcare-Associated Infections with *Pseudomonas* spp. in a Neurosurgical Hospital: Epidemiological and Clinical Insights

**DOI:** 10.3390/medicina62102019

**Published:** 2026-10-19

**Authors:** Nicoleta Luchian, Cristian Guțu, Camer Salim, Alina Pleșea-Condratovici, Marius Gabriel Dabija, Mădălina Irina Ciuhodaru, Mădălina Duceac (Covrig), Constantin Marcu, Irina Luciana Gurzu, Petruța Iuliana Moraru, Mădălina Nicoleta Matei, Letiția Doina Duceac

**Affiliations:** 1 Doctoral School of Biomedical Sciences, Faculty of Medicine and Pharmacy, “Dunărea de Jos” University of Galați, 47 Domnească Street, 800008 Galati, Romania; nicoletaluchian13@yahoo.com (N.L.); madalinaduceac@yahoo.ro (M.D.); 2 Faculty of Medicine and Pharmacy, “Dunărea de Jos” University of Galați, 47 Domnească Street, 800008 Galati, Romania; dr.c.gutu@gmail.com (C.G.); marcu_saar@yahoo.de (C.M.); iuliana.moraru@ugal.ro (P.I.M.); madalina.matei@ugal.ro (M.N.M.); letimedr@yahoo.com (L.D.D.); 3 Faculty of Medicine, Ovidius University Constanța, 900740 Constanta, Romania; 4 Faculty of Medicine, “Grigore T. Popa” University of Medicine and Pharmacy, 16 Universității Street, 700115 Iasi, Romania; mariusdabija.md@gmail.com (M.G.D.); madalina.nour@umfiasi.ro (M.I.C.); 5 Department of Preventive Medicine and Interdisciplinarity, Faculty of Medicine, “Grigore T. Popa” University of Medicine and Pharmacy, 16, Universității Street, 700115 Iasi, Romania; irina-luciana.gurzu@umfiasi.ro

**Keywords:** healthcare-associated infection, epidemiology, antibiotic resistance, *Pseudomonas*, neurosurgery

## Abstract

*Background and Objectives*: Healthcare-associated infections (HAIs) caused by *Pseudomonas aeruginosa* represent a challenge for healthcare systems worldwide, with significant incidence, high mortality, and increasing antibiotic resistance. This paper aims to describe and analyze the epidemiology of HAI cases with *Pseudomonas aeruginosa* in a tertiary neurosurgical hospital in the northeastern region of Romania during 2020–2024. *Materials and Methods*: Study inclusion criteria were patients of any age (as the hospital also admits pediatric patients from the region) and a diagnosis of *P. aeruginosa* HAI, with a signed informed consent form by the adult patient or relatives for pediatric patients. *Results*: During 2020–2024, 163 cases of *P. aeruginosa* HAIs were identified. The annual incidence decreased from 4.41‰ in 2020 to 2.37‰ in 2024 (*p* = 0.0038). Most cases occurred in the intensive care unit (ICU) (119/163; 73.0%), followed by neurosurgery wards (37/163; 22.7%). Overall, 110 cases (67.5%) occurred in men and 53 (32.5%) in women. The analysis also showed a predominance of late-onset cases and the presence of antimicrobial-resistant phenotypes among the identified *P. aeruginosa* isolates. *Conclusions*: This study provides an integrated epidemiological characterization of *P. aeruginosa* HAIs in a regional neurosurgical hospital, highlighting the predominance of ICU-associated cases, late-onset infections, and antimicrobial-resistant phenotypes. These findings support the need for continued epidemiological surveillance and antimicrobial resistance monitoring in high-risk neurosurgical settings.

## 1. Introduction

Healthcare-associated infections (HAIs) caused by *Pseudomonas aeruginosa* represent a major challenge for healthcare systems worldwide, characterized by significant incidence, high mortality, and increasing antibiotic resistance. This Gram-negative, opportunistic bacterium is capable of colonizing hospital environments and causing severe infections, particularly in vulnerable patients admitted to intensive care units or undergoing invasive procedures. Globally, data from the Centers for Disease Control and Prevention (CDC) in the United States indicate that *P. aeruginosa* is responsible for approximately 32,600 hospital-acquired infections annually, of which over 2700 result in death. In the Veterans Health Administration (VHA), a retrospective study conducted between 2009 and 2022 identified over 8000 cases of *P. aeruginosa* bacteremia, with an incidence that decreased from 4.2 to 2.6 cases per 100,000 patient-days, suggesting an improvement in control and prevention measures. However, the 30-day mortality rate remained high, hovering around 23.3% [1].

In Asia, a multicenter study conducted in China between 2017 and 2021 reported an incidence rate of *P. aeruginosa* bloodstream infections ranging from 2.37 to 3.51 cases per 100,000 patient-days. The 30-day mortality was 17.89%, and 34% of the bacterial isolates showed resistance to carbapenems, highlighting the difficulty of treatment in these cases [2].

In Europe, data provided by the European Centre for Disease Prevention and Control (ECDC) show that *P. aeruginosa* is one of the most frequent etiological agents of nosocomial infections, especially in intensive care units. The prevalence varies between 5% and 15% of all healthcare-associated infections, with a higher incidence in Southern European countries, such as Italy and Greece, where carbapenem resistance is particularly worrying [3].

In Romania, local studies indicate a significant presence of *P. aeruginosa* in university and county hospitals. A relevant example is the study conducted at the “Sf. Parascheva” Clinical Hospital of Infectious Diseases in Iași, which analyzed 62 cases of HAIs with this bacterium in 2011 and 2016 [4]. The strains were isolated from urine (50%), sputum (17.5%), wound secretions (15%), blood (10%), and central venous catheters (7.5%). Over half of the cases were nosocomial in origin, and the average length of hospital stay was 15 days [5].

Risk factors for *P. aeruginosa* infections are multiple and include both patient characteristics and elements related to the hospital environment. Prolonged hospitalization is one of the most important factors, as prolonged exposure to a contaminated environment favors colonization with resistant bacteria [6,7,8]. The use of mechanical ventilation is frequently associated with the occurrence of nosocomial pneumonia, as the biofilm formed on endotracheal tubes protects the bacteria from the action of antibiotics and the immune system [8,9,10].

Invasive medical devices, such as urinary catheters, central venous catheters, or gastric tubes, represent entry points for bacteria, and patients with chronic conditions—such as diabetes mellitus, chronic obstructive pulmonary disease (COPD), or renal failure—have compromised immunity, which makes them more susceptible to severe infections [11,12]. Patients with burns or open wounds are also at increased risk, as damaged skin no longer provides an effective barrier against pathogens [13].

Though the location of *P. aeruginosa* infections is variable, the most common forms include ventilator-associated pneumonia, catheter-associated urinary tract infections, surgical site infections, central venous catheter-associated bacteremia, and skin and soft tissue infections [14,15].

Microbiologically, *P. aeruginosa* is a Gram-negative aerobic bacterium with a remarkable ability to produce biofilms—a protective structure that adheres to medical surfaces and protects the bacteria from the actions of antibiotics and the immune system. *P. aeruginosa* strains are characterized by a natural resistance to many antibiotics, including beta-lactams, and can develop additional resistance mechanisms, such as the production of beta-lactamases, active efflux, and modification of molecular targets. Antibiotic resistance is one of the greatest challenges in the treatment of *P. aeruginosa* infections. Recent studies have shown an alarming increase in multidrug-resistant (MDR) strains, especially to carbapenems, cephalosporins, and fluoroquinolones. In the Chinese study, 34% of *P. aeruginosa* isolates were carbapenem-resistant, and 26% were classified as multidrug-resistant [16,17]. In Europe, carbapenem resistance is more common in the south of the continent, and in Romania, local data indicate an increased prevalence of resistant strains in large hospitals, especially in intensive care units. The World Health Organization (WHO) has classified *P. aeruginosa* as a “critical priority” pathogen on its global list of bacteria for which the development of new antibiotics is urgently needed. *P. aeruginosa* HAIs in neurosurgery is a particularly serious clinical problem, with major implications for the postoperative outcome, length of hospital stay, and patient prognosis. Due to the invasive nature of neurosurgical interventions and the vulnerability of the central nervous system, these infections can have devastating consequences, especially when caused by antibiotic-resistant strains. In the neurosurgical context, *P. aeruginosa* is frequently involved in surgical wound infections, postoperative meningitis, ventriculitis, and infections associated with implantable devices. The risk of infection is increased in emergency interventions, open head trauma, re-interventions, and in the presence of comorbidities, such as diabetes mellitus, immunosuppression, or pre-existing colonization with *P. aeruginosa* [18].

Postoperative meningitis with *P. aeruginosa* is one of the most severe forms of infection in neurosurgery. It usually occurs following intraoperative contamination or secondary to a cerebrospinal fluid fistula. Symptoms include fever, severe headache, altered state of consciousness, meningeal signs, and sometimes seizures. Diagnosis is based on cerebrospinal fluid analysis, which reveals a neutrophil-predominant pleocytosis, hypoglycorrhagia, and hyperproteinorrhagia, and culture identifies *P. aeruginosa* as the etiological agent [19,20].

The prevention of HAIs in neurosurgery is essential and involves strict adherence to aseptic protocols, judicious use of prophylactic antibiotics, monitoring of preoperative bacterial colonization, and careful management of implantable devices. The training of medical personnel and implementation of rigorous HAI control policies are also crucial [21,22,23,24,25,26].

HAIs with *P. aeruginosa* in neurosurgery are rare but extremely severe, requiring a multidisciplinary, rapid, and aggressive approach [27]. The prognosis depends on the promptness of diagnosis, the sensitivity of the bacteria to antibiotics, and the ability of the medical team to control the source of infection. In the era of antimicrobial resistance, these cases highlight the importance of epidemiological surveillance and continuous research in the field of anti-infective therapy [28,29].

*Aim and objectives*. This paper aims to describe and analyze the incidence and distribution of healthcare-associated infections (HAIs) with etiology represented by *Pseudomonas* strains in the wards of tertiary neurosurgical hospital in the northeastern region of Romania, over a period of 5 years (2020–2024). The objectives were to determine the incidence of HAIs caused by *Pseudomonas* strains; socio-demographic analysis of cases with particularization according to the environment of origin, sex, and age groups of patients; analysis of the hospitalization period from admission to the onset of HAIs; and evaluation of antibiotic resistance profiles of *Pseudomonas* strains.

## 2. Materials and Methods

### 2.1. Study Design

The “Prof. Dr. Nicolae Oblu” Emergency Clinical Hospital in Iași, Romania, is a 321-bed university clinical hospital that specializes in neurosurgery (NC1, NC2, and NC3), but it also includes neurology (N1 and N2), ophthalmology, neuro-ophthalmology, intensive care (ICU), radiology, neuroimaging, functional examinations and specialized laboratories. For the period of 2020–2024, the data showed that the total number of hospitalized patients was 42,851, and the total number of HAIs was 1653 cases (3.85% of all hospitalized patients).

### 2.2. Information Resources

In conducting this study, information extracted from medical documents (observation sheets, medical registers, HAI files, statistical reports, HAI surveillance registry and hospital control service) was used.

*Study inclusion criteria*: Patients of any age (as the hospital also admits pediatric patients from the region; pediatric hospitals currently do not have a pediatric neurosurgery department); a diagnosis of *P. aeruginosa* HAI; and a signed informed consent form by the adult patient or relatives for pediatric patients.

The diagnosis of HAI with *P. aeruginosa* was made by taking into account the microbiological result of the culture of the pathological product (tracheal aspirate; cerebrospinal fluid; secretion from the surgical wound/drain tube; blood culture; secretions from the superficial pressure ulcer; urine culture; and skin secretion at the catheter implantation site/catheter tip culture). As a time interval, they were collected as follows: at least 72 h after hospitalization if clinical signs appeared, or for patients with ventilator-associated pneumonia (VAP), and if the patient with pneumonia presented with an invasive respiratory device, even temporarily; and 48 h before the onset of pneumonia—for superficial surgical site infections, the infection was considered to have occurred within a maximum of 30 days after surgery.

HAI cases were identified based on positive microbiological cultures from pathological biological products collected from the patients and recorded in the hospital HAI surveillance records.

*Exclusion criteria*: Patients who did not present with a diagnosis of HAI with *P. aeruginosa*; and those who did not sign the informed consent form.

### 2.3. Antibiotic Susceptibility Testing

The microtablet kits used were chosen by the laboratory bacteriologist depending on the tested germ (Gram-positive or Gram-negative), its natural resistance, the location of the infection, and the tested product, in accordance with CLSI for the diffusimetric method for performing antibiograms and based on the commercialization of the antibiotic in pharmacies in Romania—the following rules were adhered to: imipenem and meropenem were used for urine cultures with *Pseudomonas*; netilmicin was used only for *Pseudomonas*, regardless of the pathological product (urine, wound secretion, tracheal aspirate, catheter tip, etc.); ceftazidime/avibactam was used only for *Pseudomonas*, regardless of the product and for *Enterobacteriacaeae* MDR; and aztreonam was used for *Pseudomonas* and *Enterobacteriacae*, regardless of the pathological product and bacteria. In this study, MDR was defined as non-susceptibility to at least one agent in three or more antimicrobial categories.

Examples of antimicrobial agents included in the susceptibility testing of Pseudomonas isolates were piperacillin/tazobactam, ceftazidime, ceftazidime/avibactam, cefepime, aztreonam, imipenem, meropenem, netilmicin, tobramycin, ciprofloxacin, levofloxacin, amikacin, and gentamicin. Colistin susceptibility was assessed by MIC determination in selected isolates.

The emergence, spread, and selection of antibiotic-resistant bacteria represent a real threat to patients admitted to the “Prof. Dr. N. Oblu” Emergency Clinical Hospital in Iași, given that infections with antibiotic-resistant bacteria result in increased morbidity and mortality among patients, as well as an increased length of hospitalization and implicitly increased costs; antibiotic resistance frequently leads to a delay in appropriate antibiotic treatment.

Each patient contributed one HAI episode during the study period, with one positive microbiological result from a single clinical site; no repeated admissions or duplicate episodes were identified.

Prudent use of antibiotics can prevent the emergence and selection of antibiotic-resistant bacteria; in this regard, antibiotic resistance analysis in the hospital is considered appropriate. The pathological products from which microbial strains responsible for infections were isolated were: urine, surgical/non-surgical wound secretions, pharyngeal and nasal exudates, catheter tip, drain tube, conjunctival secretions, ear secretions, tracheal aspirate, eschar, abscess pus, CSF/CSF fistula, and blood cultures. A wide range of antibiotic batteries (e.g., fluoroquinolones, cephalosporins, carbapenems, amicoglycosides, etc.) were used to perform the antibiograms.

### 2.4. Statistical Analysis

The information material was processed according to various socio-demographic, etiological, clinical, and epidemiological parameters, and the results were interpreted using classical epidemiological, statistical–mathematical, and computerized methods. Patients’ data were anonymized and collected in Microsoft Office Excel before being processed.

Incidence rates were calculated based on the number of HAI cases and the total number of patients discharged for each year. Temporal trends were assessed using simple linear regression in Microsoft Excel, and R^2^ values were used to describe the fit of the linear model. All analyses were exploratory and descriptive. For the comparison of annual incidence values, the *p*-values shown in Table 1 were obtained using a simple linear regression model (slope significance test).

### 2.5. Ethical Considerations

Formal approval for the study was obtained from the Hospital Ethics Committee, and the general provisions of the Declaration of Helsinki on Medical Research Involving Human Subjects were respected. Data collection began by searching the health unit database and selecting cases of patients who contracted HAIs during hospitalization.

The identification of HAI cases was performed by active or passive screening, according to case definitions based on clinical, epidemiological, and laboratory considerations, as specified in the Order of the Ministry of Health 1101/2016 with reference to Decision 2012/506/EU.

## 3. Results

The incidence of HAIs with *Pseudomonas* spp. during 2020–2024 showed a moderate increase, ranging between 0.207% and 0.430%, with an average value of 0.380%. This evolution reflects a relatively stable circulation of the pathogen within the hospital environment, without major fluctuations and without obvious epidemic episodes, which denotes epidemiological control in accordance with hospital norms and legislation in force. Although the linear trend was statistically significant (*p* = 0.0038), the year-to-year variations were small and gradual, without abrupt increases or epidemic episodes (Table 1).

The burden of HAIs with *Pseudomonas* spp. in the hospital remains relatively low compared to aggregate European levels, suggesting effective transmission control and lower ecological pressure for this pathogen.

### 3.1. Distribution by Wards

The distribution of HAI cases reported by the wards that were analyzed during the period of 2020–2024 highlights a majority concentration in the intensive care unit (ICU). Of the total 163 cases identified in these wards, 119 (73.0%) came from the ICU, while the neurosurgery wards (NC1 and NC2) accounted for 37 cases (22.7%), and the neurology wards (N1 and N2) only had seven cases (4.3%). This distribution is consistent with the epidemiological profile of critically ill patients, in whom exposure to invasive devices, severity of pathologies, and prolonged duration of hospitalization significantly increase the risk of infection [11]. Analysis of the temporal evolution shows a progressive increase in the number of cases reported in the studied departments, from 12 cases in 2020 to 50 cases in 2024. However, this increase is not proportional to the dynamics of HAIs at the hospital level, where the values vary between 244 and 334 cases annually. The share of the analyzed departments in the total HAI cases increases constantly, from 4.9% in 2020 to 15.0% in 2024. The differences between neurosurgery departments (NC1/NC2) may reflect variations in casuistry, type of interventions, or clinical practices, with NC2 reporting a higher number of cases (21 compared to 16). Neurology departments maintain low values throughout this period, consistent with the non-invasive profile of patients and the low epidemiological risk that is characteristic of these departments.

**Figure 1:**
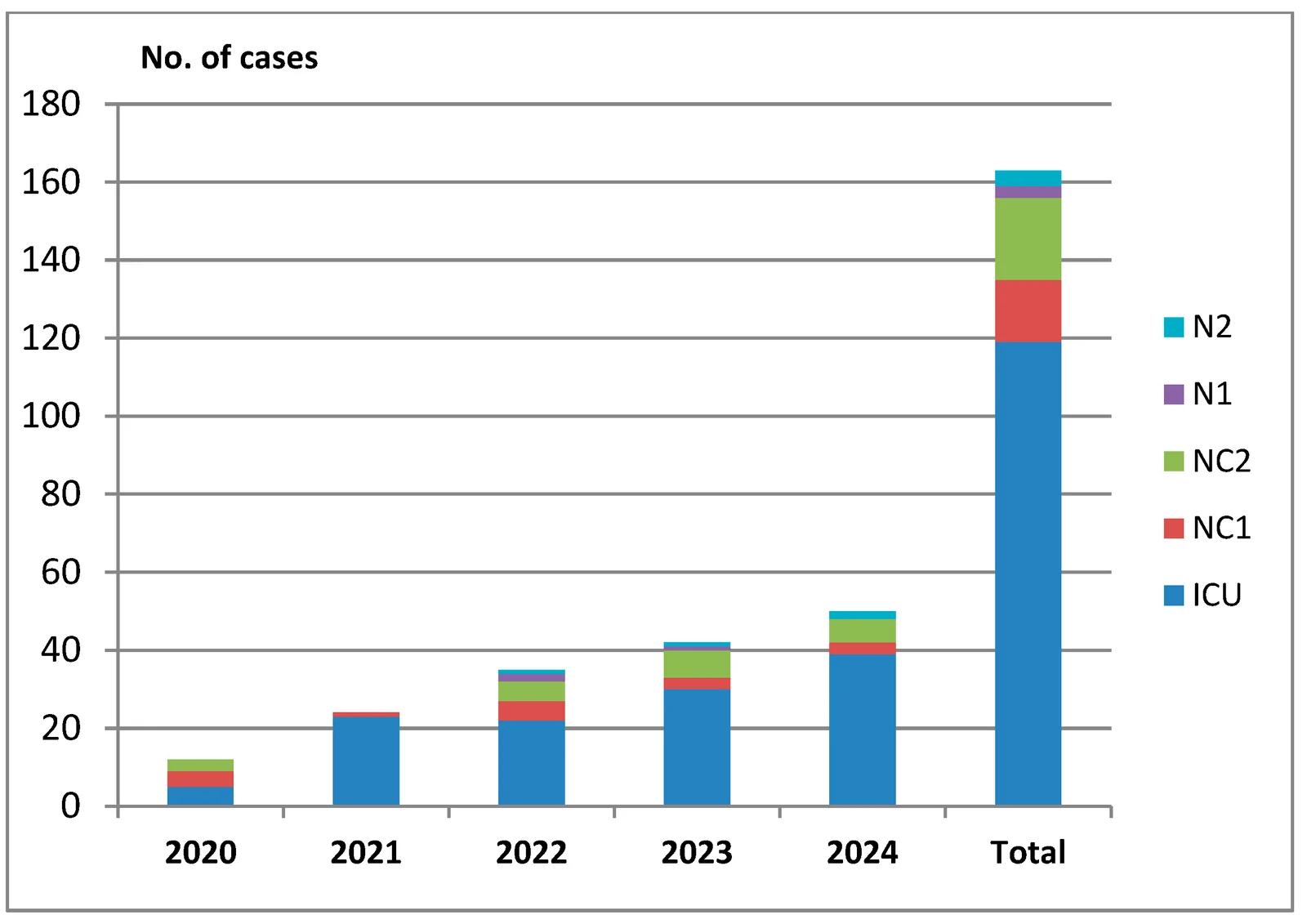
Distribution of HAI isolates positive for *P. aeruginosa* by wards (NC1, NC2—neurosurgery departments; N1, N2—neurological departments; ICU—intensive care unit) and by study years compared to the total number of cases.

The ICU department represents the main determinant of the burden of HAIs in the hospital, and the increase in reporting in the post-pandemic period reflects the strengthening of epidemiological surveillance while emphasizing the need to maintain HAI control interventions in ICUs and differentiated monitoring by departments, with a focus on high-risk areas (Figure 1).

**Figure 2:**
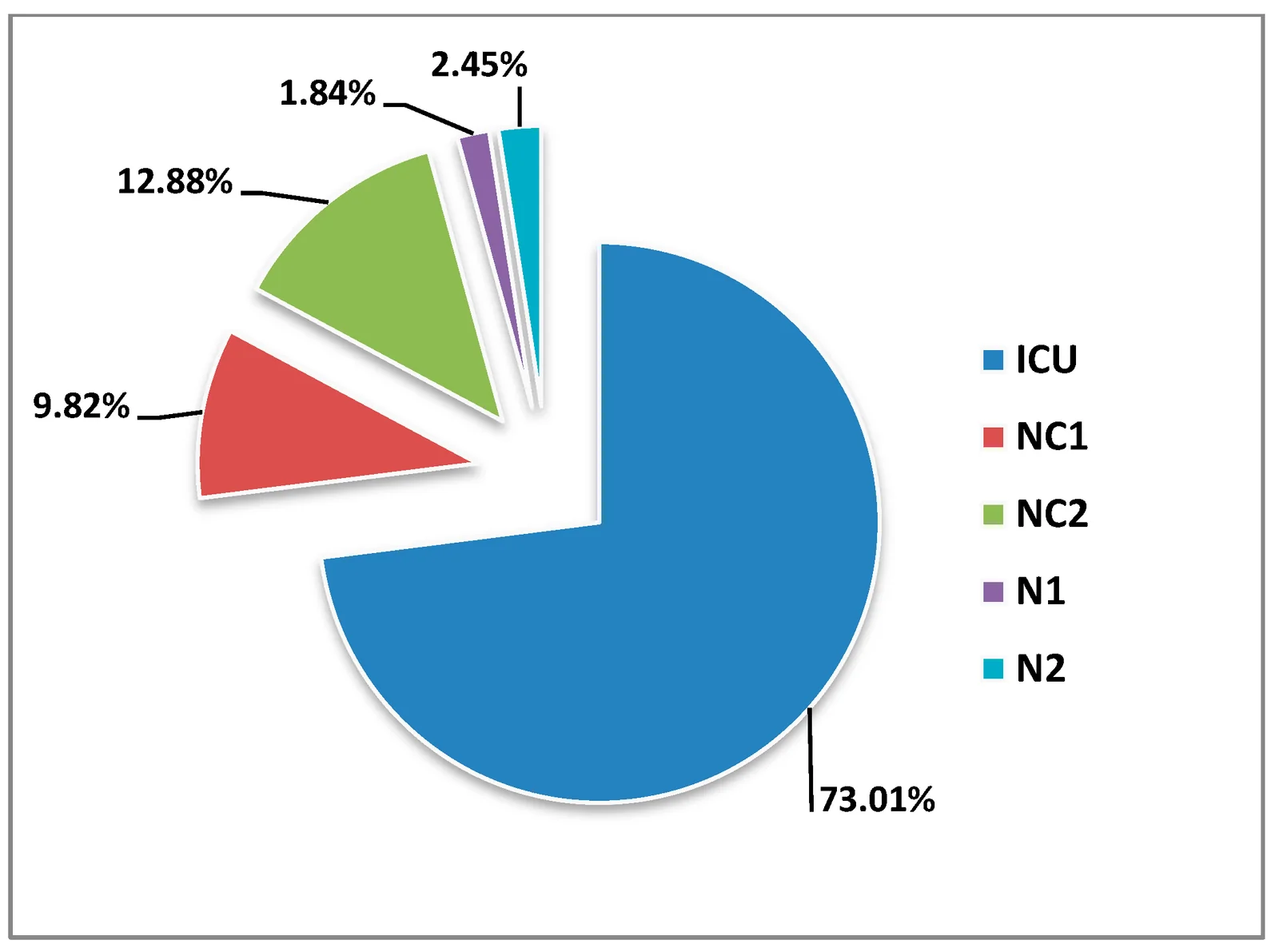
Distribution of HAI isolates positive for *P. aeruginosa* by wards (NC1, NC2—neurosurgery departments; N1, N2—neurological departments; ICU—intensive care unit).

The ICU contribution to the total number of HAI cases was dominant throughout the period analyzed. The proportion of cases originating from the ICU ranged from 41.7% in 2020 to 95.8% in 2021, subsequently remaining within a range of 62.9–78.0% in 2022–2024. The ICU generated 73.0% of the total HAI cases reported in the studied wards. This distribution confirms the central role of intensive care in the dynamics of HAIs, reflecting the profile of critically ill patients, increased exposure to invasive devices, and prolonged length of hospital stay, all of which are factors that amplify the risk of colonization and infection (Figure 2).

### 3.2. Distribution by Demographic Characteristics

**Figure 3:**
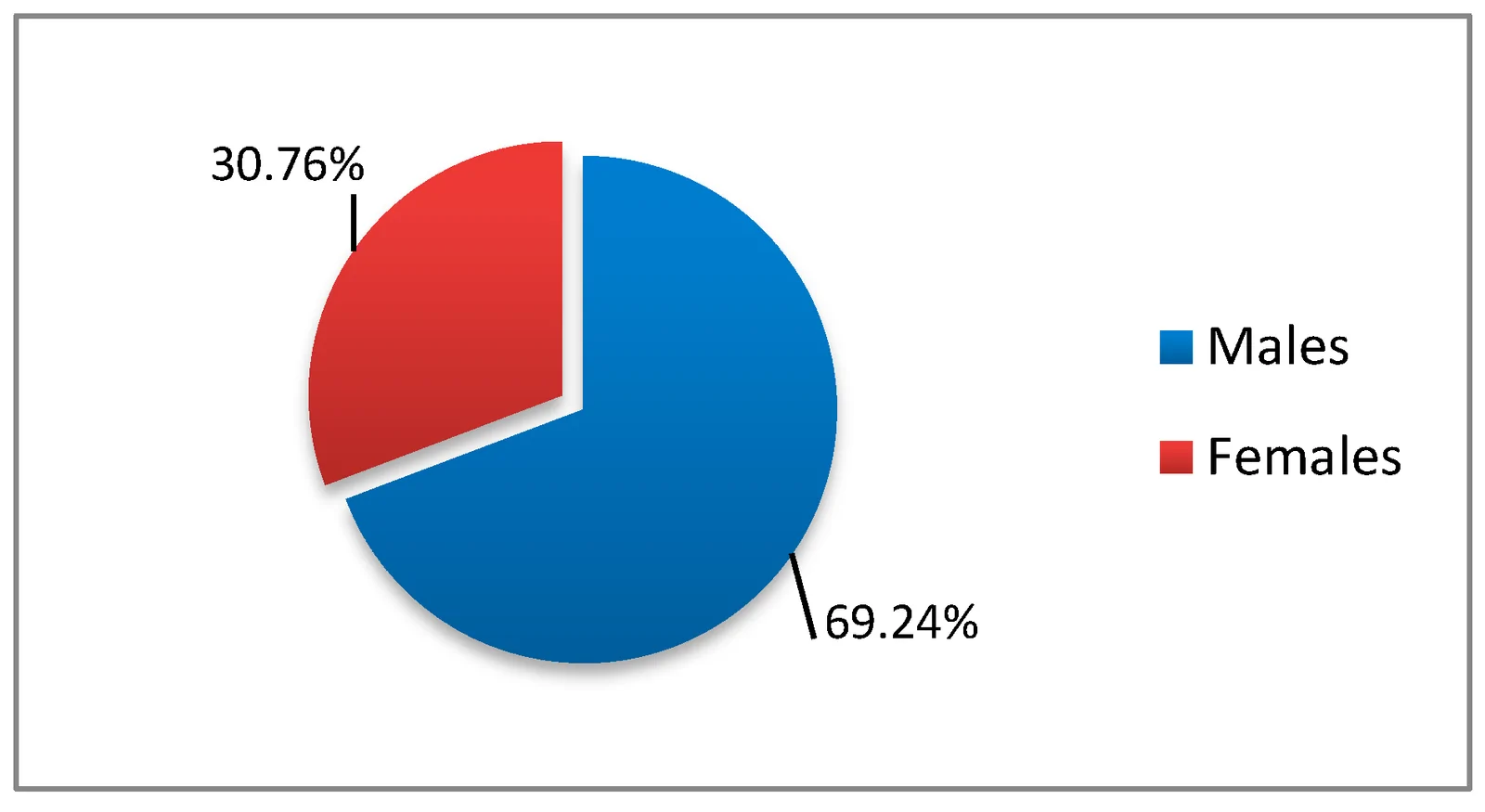
Distribution of HAI cases with *P. aeruginosa*, differentiated by gender.

**Figure 4:**
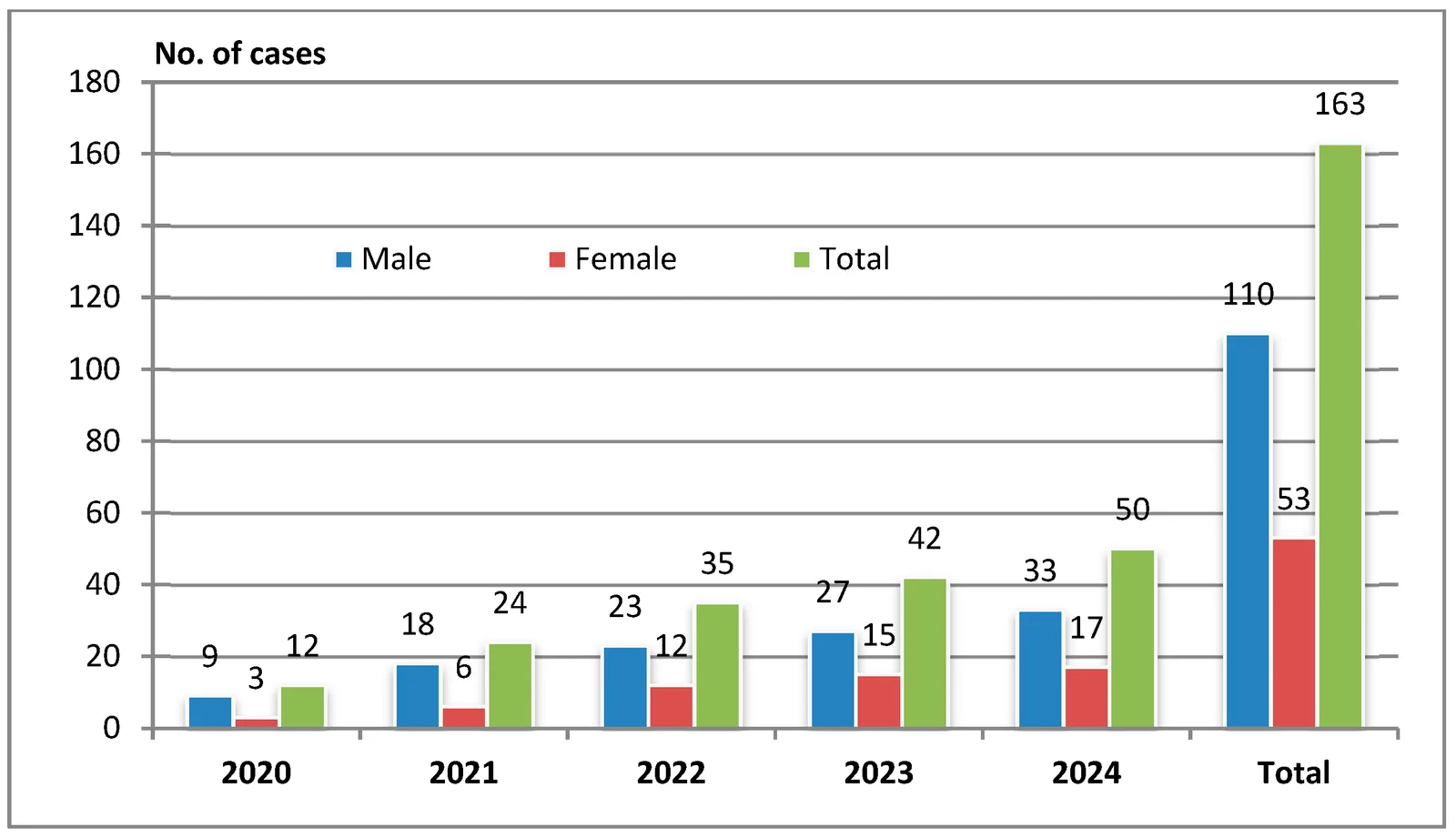
Evolution of HAI cases with *P. aeruginosa* during the period of 2020–2024, differentiated by gender and annual totals.

The gender distribution of HAI cases showed a M:F ratio = 2.075 (110 cases in males—67.48%; 53 cases in females—32.52%) (Figure 3). A steady increase in the number of cases was observed from one year to the next in both men and women. Throughout the analyzed period, men consistently presented a higher number of cases of HAIs with *P. aeruginosa* compared to women. The difference is evident in each year; for example, in 2024 there were 33 cases in men compared to 17 in women. In total, of the 163 reported cases, 110 were in men and 53 in women. Thus, approximately two thirds of the cases were found in men, as can be seen from the M:F ratio (Figure 4).

The progressive increase in the total number of cases may reflect several factors, such as improved surveillance and reporting of HAIs, an increase in the number of hospitalized patients, or the presence of more frequent risk factors among male patients. Incidence values already incorporate annual variations in admissions, as they were calculated per the number of patients discharged.

**Figure 5:**
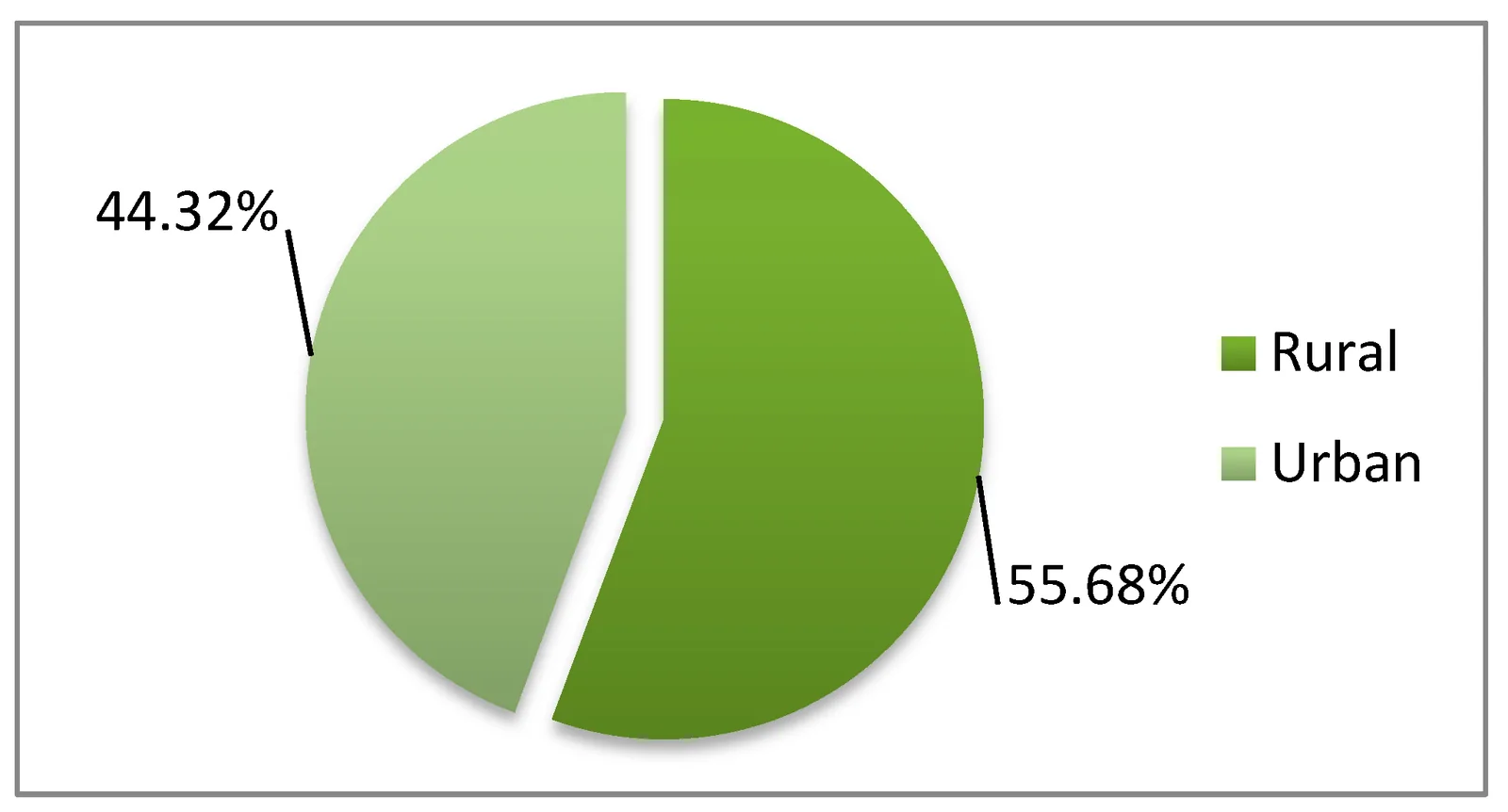
Distribution of HAI cases positive for *P. aeruginosa* by areas of residence.

Depending on the areas of residence of patients who developed HAIs with *P. aeruginosa*, 88 patients, or 53.98%, came from urban areas, and 55 patients, or 46.02%, from rural areas, with a U:R ratio = 1.17 (Figure 5).

**Figure 6:**
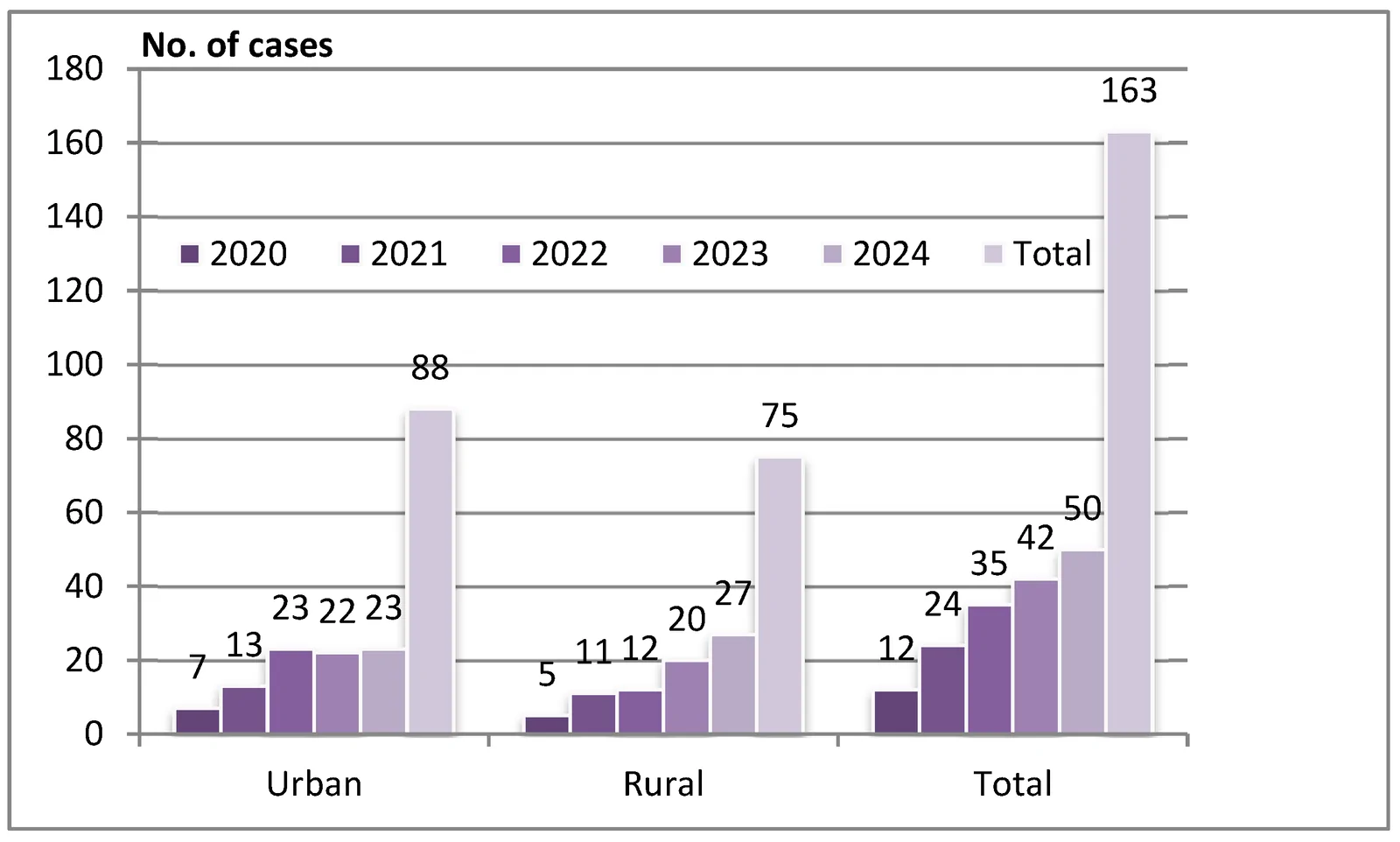
Distribution of HAI cases positive for *P. aeruginosa* by areas of residence and by year during 2020–2024.

In 2020, the fewest cases were recorded, with seven cases in urban areas and five in rural areas. In 2023, 22 cases were reported in urban areas and 20 in rural areas. The only year in which rural areas exceeded urban areas in terms of the number of cases was 2024 (23 cases in urban areas and 27 in rural areas). This finding may indicate an expansion of risk factors and exposure to HAI cases in the rural population, as well as an increased addressability of patients from these areas to specific regional and university health units (Figure 6).

Regarding the distribution of HAI cases with *P. aeruginosa* by age group, during the studied period, we showed that the frequency of cases increased with age, reaching a maximum in the 45–64 year category, after which it registered a slight decrease in the 65–84 year group. The most affected age group was represented by patients aged 45–64 years, with 80 cases, followed by the 65–84 year group, with 71 cases, which could indicate an increased vulnerability of adults and the elderly to HAIs with *P. aeruginosa* (Table 2).

**Figure 7:**
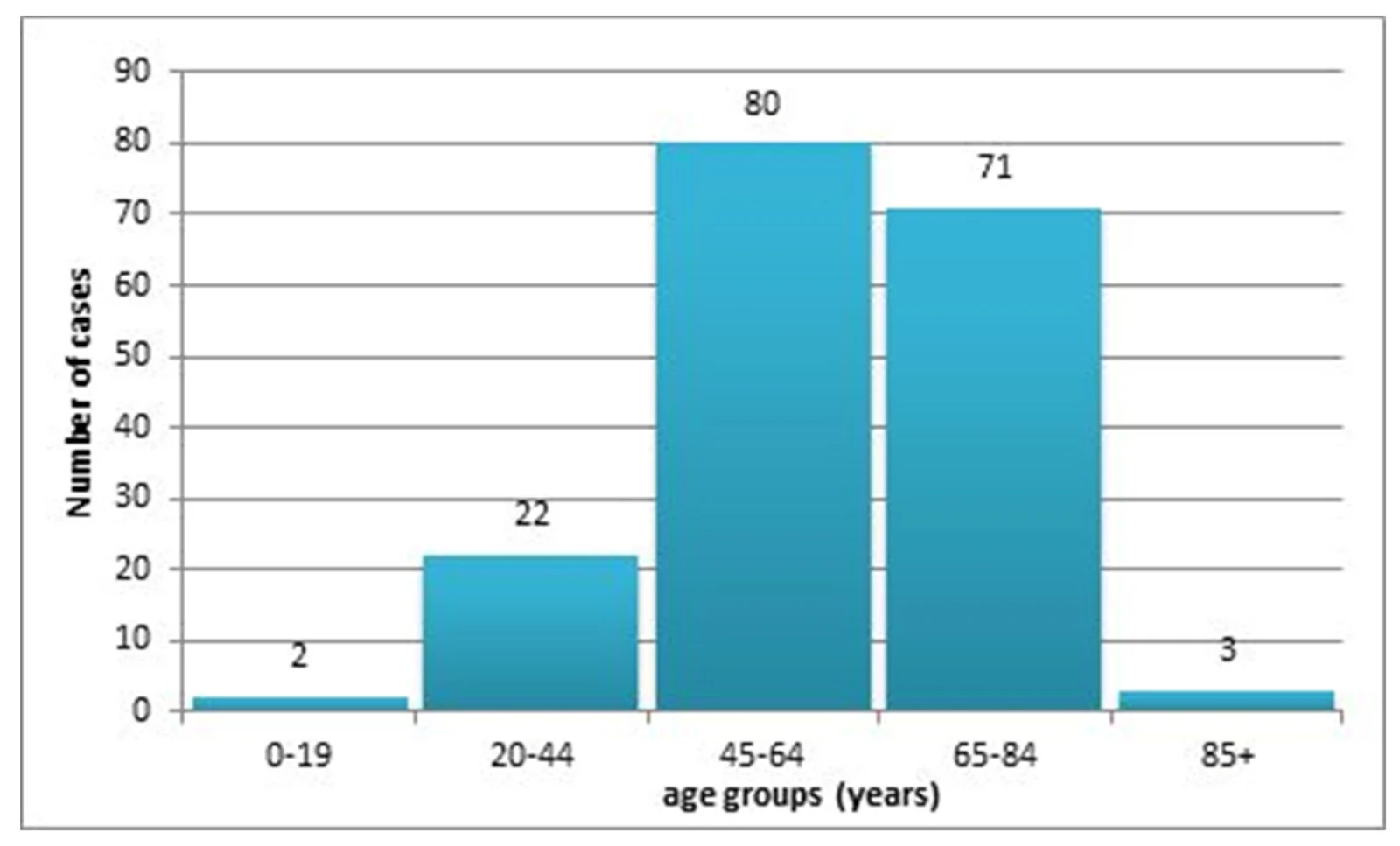
Distribution of cases per age group in years, highlighted by the histogram of age.

The linear regression indicates a general trend of increasing the number of cases with advancing age, but the low value of the coefficient of determination (*R*^2^ = 0.0464) shows that the relationship between age and the number of cases is not a strong linear one. This aspect suggests that the occurrence of HAIs with *P. aeruginosa* is also influenced by various factors, such as comorbidities, length of hospitalization, invasive procedures, or the immunological status of patients (Figure 7).

### 3.3. Hospitalization Days

**Figure 8:**
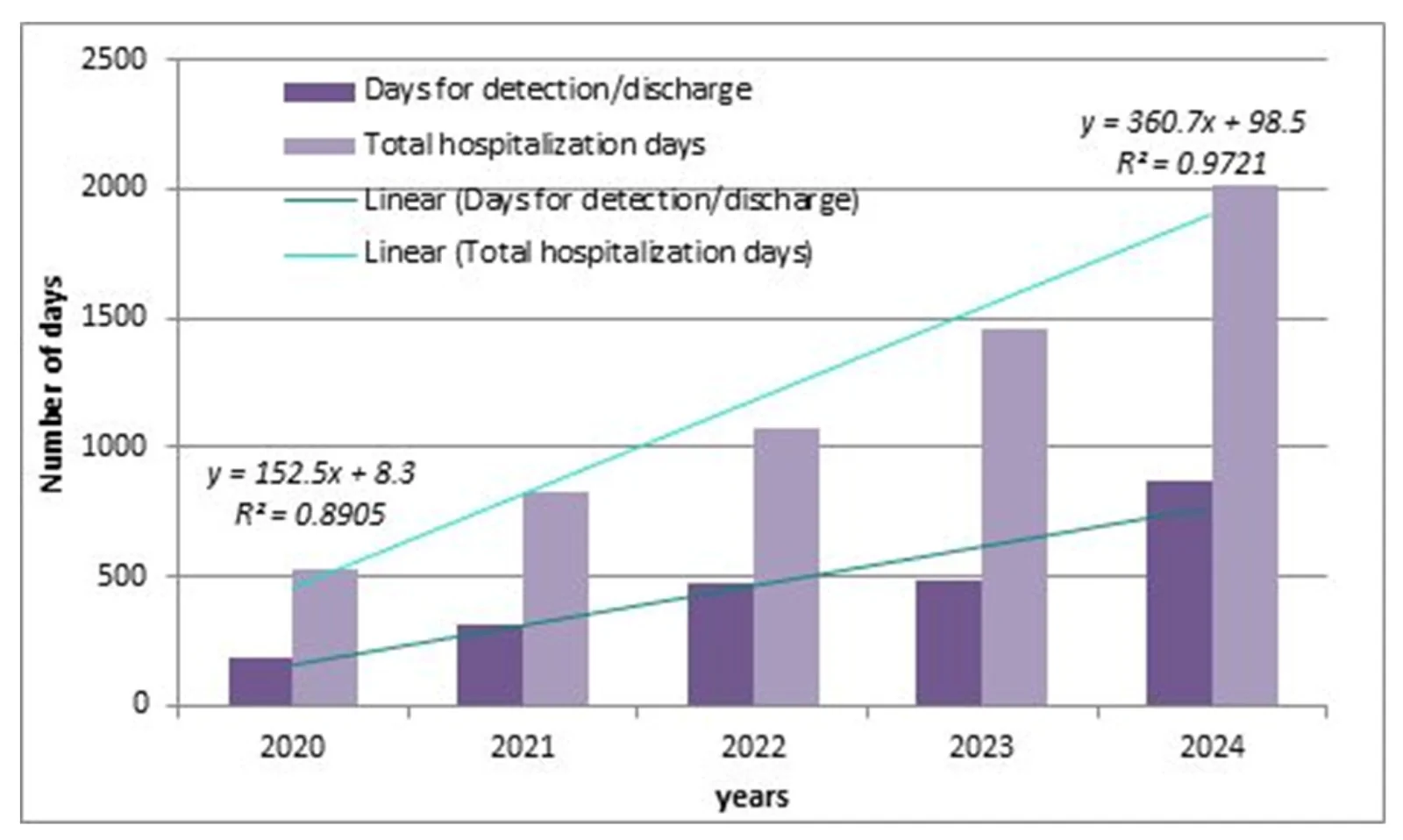
Days of hospitalization of HAI cases with positive isolates for *P. aeruginosa* (R^2^ or R squared—coefficient of determination; x—independent variable; y—dependent variable, from which *R*^2^ shows how much of the variation in the dependent variable y is explained by the independent variable x).

The total number of hospitalization days increased steadily during 2020–2024, from approximately 1000 days in 2020 to over 2000 days in 2024, representing an increase of approximately 110% over the study period. The interval between hospitalization and the detection of infection followed a similar upward trend, increasing from approximately 150 cumulative days in 2020 to over 750 days in 2024, representing an increase of approximately 400%. Both series showed a strong temporal correlation with stable linear trends (R^2^ = 0.89 for days to detection and *R*^2^ = 0.97 for total hospitalization days), indicating a progressive and sustained evolution of the duration of hospitalization and the time required to identify HAIs (Table 3, Figure 8).

**Figure 9:**
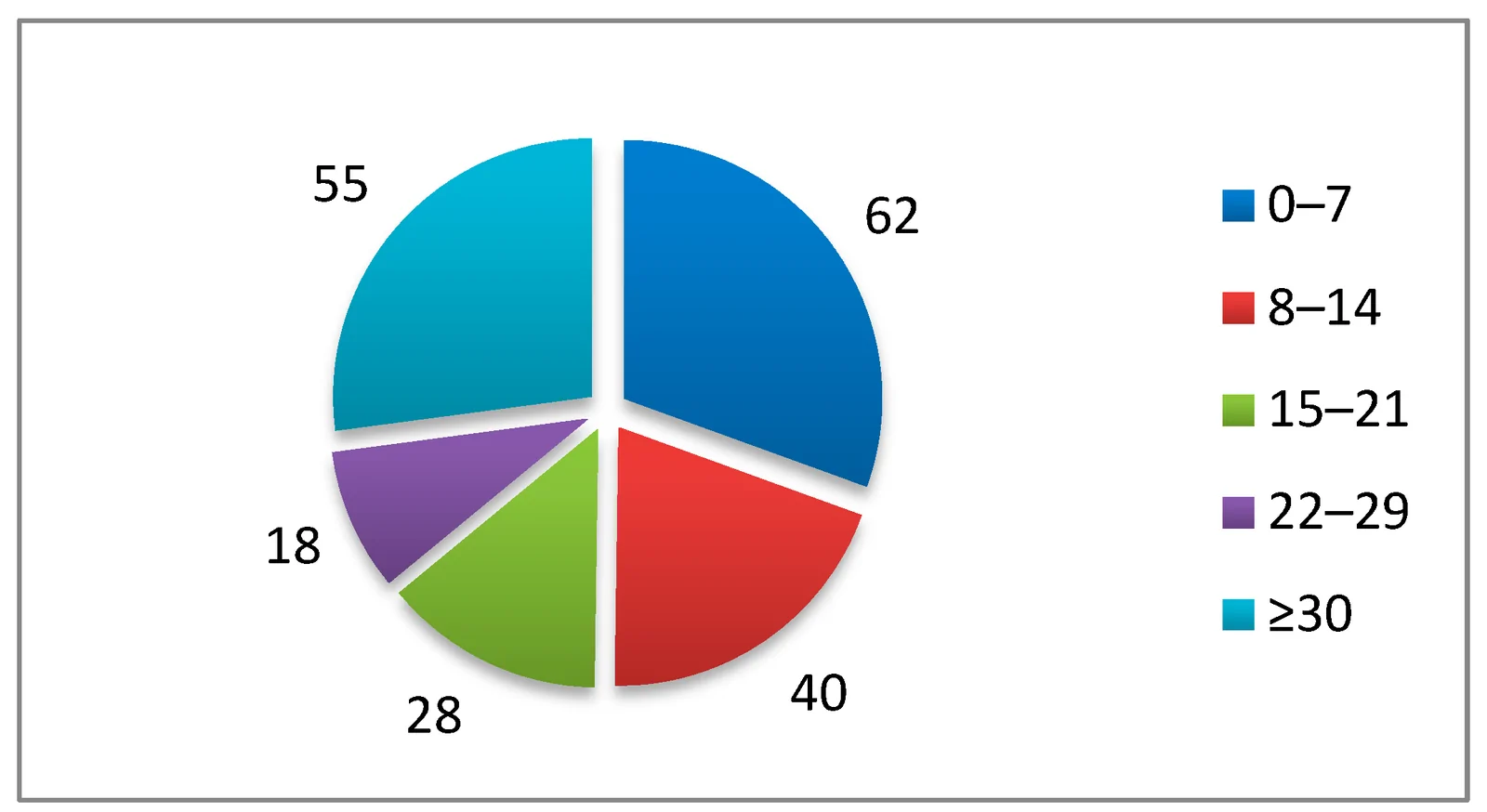
Distribution of the number of patients by hospitalization day intervals (from detection to discharge).

We grouped these intervals into intervals of 0–7 days, 8–14 days, 15–21 days, 21–29 days, and more than 30 days. Cumulative analysis of the interval between hospitalization and the detection of infections revealed a heterogeneous distribution, with a predominance of early and late forms. The interval 0–7 days was the most frequent, with 62 cases (38.0%), followed by the interval ≥ 30 days, with 55 cases (33.7%). HAIs detected between 8 and 14 days represented 40 cases (24.5%), whereas those in the intervals of 15–21 days and 22–29 days amounted to 28 cases (17.2%) and 18 cases (11.0%), respectively. This distribution shows that over two thirds of the infections were either identified early or after prolonged periods of hospitalization (Figure 9).

### 3.4. Clinical Entities

During the period of 2020–2024, 163 cases of HAIs with *P. aeruginosa* were reported and heterogeneously distributed between the main clinical entities. Respiratory forms predominated, with 71 cases of ventilator-associated pneumonia (VAP, 43.6%) and 37 cases of lower respiratory tract infections (LRTIs, 22.7%). Urinary tract infections totaled 25 cases (15.3%), and surgical site infections in 13 cases (8.0%). Pressure ulcers were associated with nine cases (5.5%), and post-neurosurgical meningitis with eight cases (4.9%).

**Figure 10:**
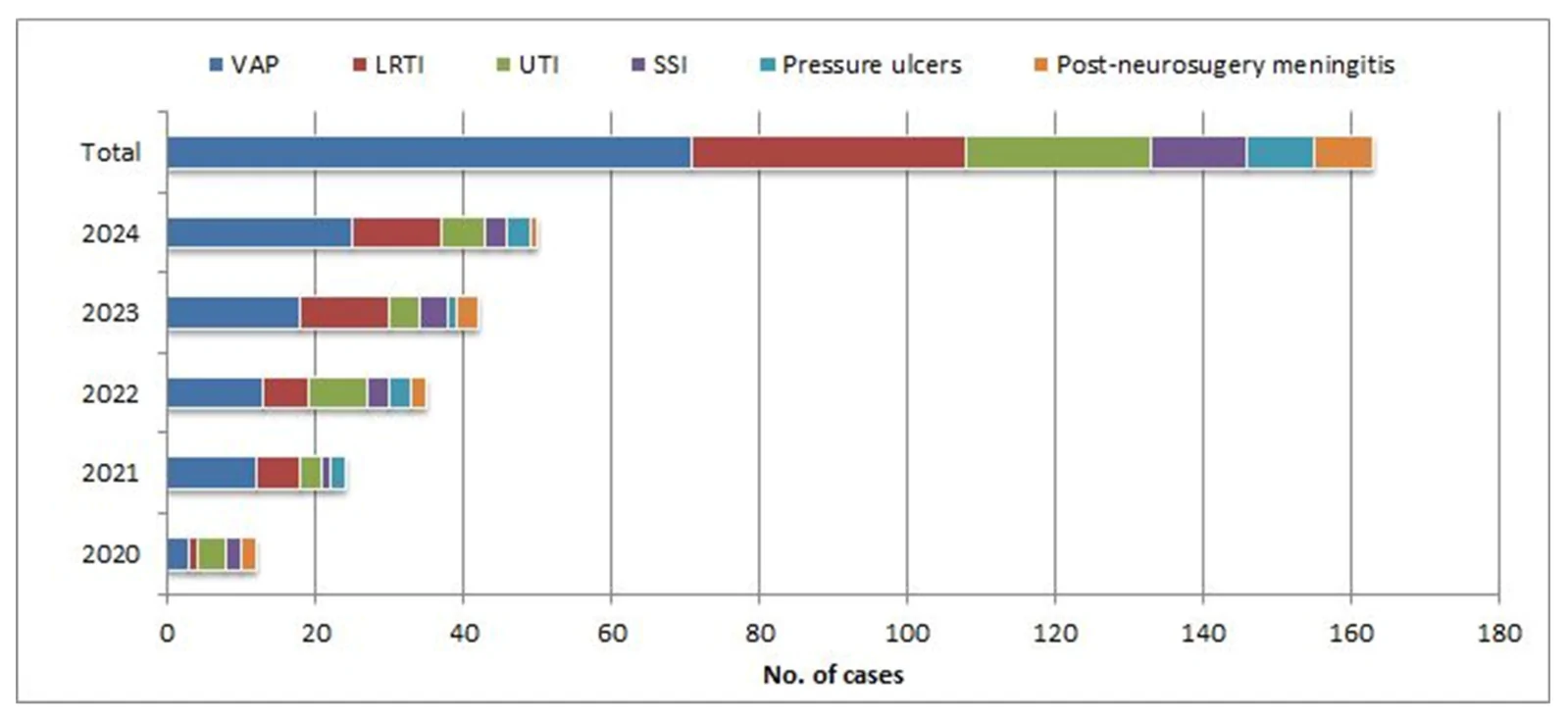
Proportion of clinical entities of *Pseudomonas*-positive isolates (VAP—ventilator-associated pneumonia; LRTI—lower respiratory tract infection; SSI—surgical site infection; UTI—urinary tract infection).

The annual distribution of these clinical entities showed a progressive increase in the number of cases of respiratory forms, especially VAP, which became dominant in the last few years of the study period (Figure 10).

### 3.5. Antimicrobial Resistance

For the purposes of this study, antimicrobial resistance profiles were classified into the following categories: carbapenem-resistant (CR), multidrug-resistant (MDR), CR/MDR, ESBL-producing with carbapenem resistance (ESBL+CR), and not available (N/A). CR and CR/MDR were considered distinct, mutually exclusive categories. The resistance categories were assigned based on the antimicrobial susceptibility testing results available for each isolate.

**Figure 11:**
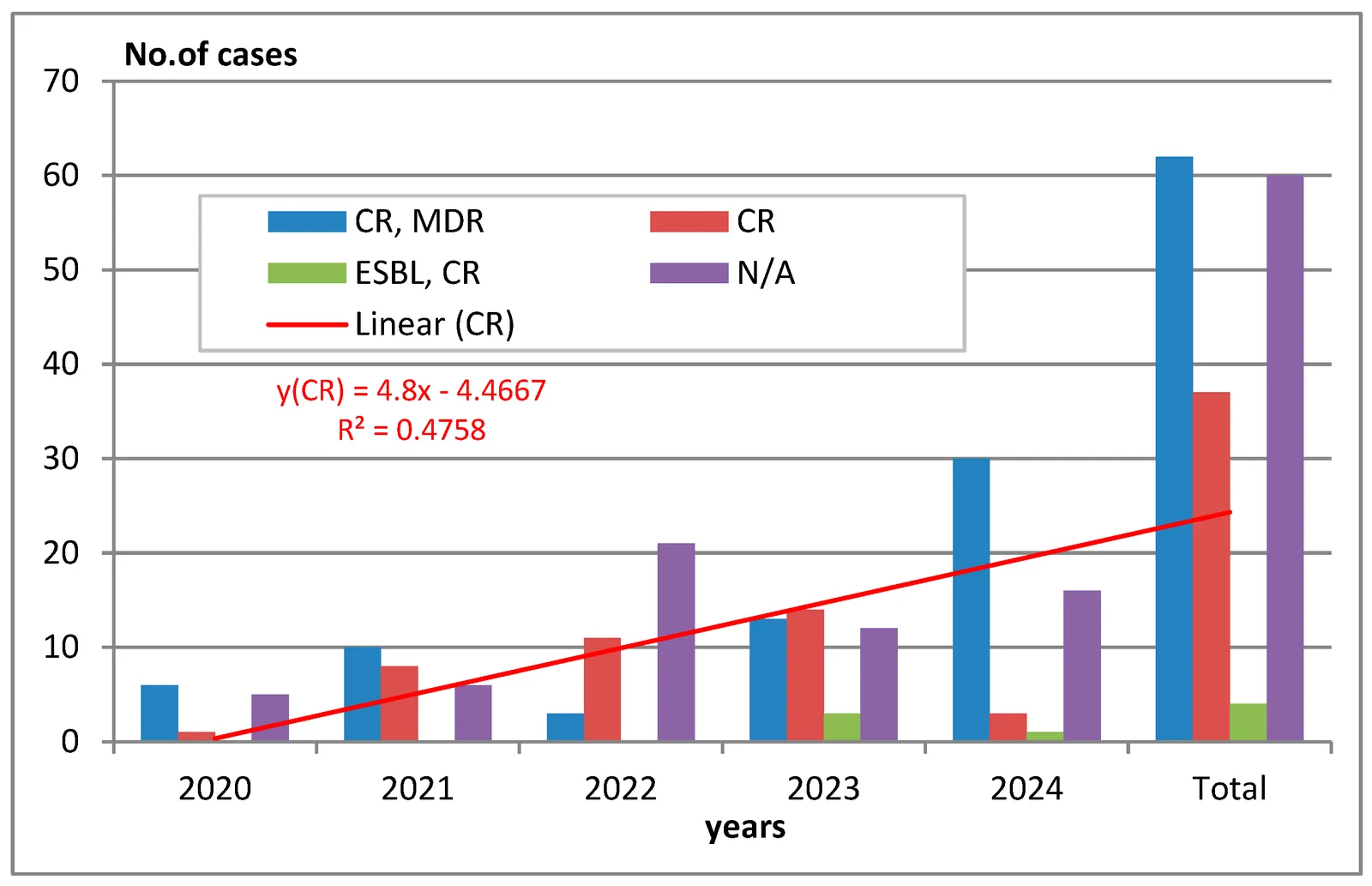
Types of antimicrobial resistance of *Pseudomonas* strains (CR—carbapenem-resistant; MDR—multidrug-resistant; ESBL—extended-spectrum beta-lactamase; NA—not available) (*R*^2^ or R squared—coefficient of determination; x—independent variable; y—dependent variable, from which *R*^2^ shows how much of the variation in the dependent variable y is explained by the independent variable x).

The pathogenicity criterion, in terms of antimicrobial resistance, was investigated by analyzing the results of microbial cultures, as recorded and processed in the hospital microbiology laboratory. Analysis of the resistance profiles for 163 *P. aeruginosa* isolates showed a predominance of carbapenem-resistant and multidrug-resistant (CR/MDR) strains, which totaled 62 cases (38.0%). Carbapenem-resistant (CR) strains alone accounted for 37 cases (22.7%), with a marked annual variation and moderate upward trend. ESBL (extended-spectrum beta-lactamase) phenotypes associated with carbapenem resistance were rare, having been identified in four cases (2.5%). For 60 isolates (36.8%), resistance data were not available. The annual distribution highlights an important increase in CR/MDR strains in 2024 (30 cases), which contributes to the recent change in the overall resistance (Figure 11).

## 4. Discussion

The results of our study highlight a complex epidemiological profile of healthcare-associated infections with *P. aeruginosa*, as characterized by annual variations in the number of cases, heterogeneous distributions of detection intervals, and relevant demographic differences. These elements must be interpreted in the particular context of antimicrobial resistance, given that *P. aeruginosa* is one of the main bacteria involved in HAIs with MDR and carbapenem-resistant phenotypes. The presence of these phenotypes influences both the severity of the clinical course, the duration of hospitalization, and the likelihood of late infections, contributing to a persistent infection risk among patients with prolonged hospitalizations. In this context, the dynamics of detection intervals and the distribution of cases by gender and residence environment reflect the interactions between individual patient factors, the complexity of medical procedures, and the selective pressure exerted by antibiotic consumption at the institutional level.

The incidence rate values obtained are below the ranges reported in the European literature, where the overall rates of HAIs vary approximately between 0.2 and 0.6%, depending on the patient mix, the complexity of the interventions, and the presence of an intensive care unit [3,4].

The distribution of cases by gender and residence environment completes the epidemiological profile of patients affected by HAIs with *P. aeruginosa*. The predominance of cases in men (M:F ratio = 2.07:1) is consistent with data from the specialized literature, where men frequently present a higher prevalence of comorbidities and risk factors associated with severe infections [30,31]. Analysis of the environment of origin shows a slight predominance of patients from urban areas (53.98%), but the annual dynamics reveal significant variations. We observed that in 2024, the rural area exceeded the urban area for the first time (27 vs. 23 cases), suggesting not just a possible extension of risk factors to the rural population but also an increased addressability of patients from these areas to regional and university health units. In the context of a hospital with a neurology and neurosurgery profile, this change emphasizes the need for continuous monitoring of the geographical distribution of HAIs, as changes in the access to medical services, case severity, and patient flows can significantly influence local epidemiology [32,33].

The distribution of HAI cases across hospital wards analyzed highlights a clear risk gradient, with intensive care representing the main determinant of HAI burden. The high proportion of cases originating from ICUs reflects cumulative exposure to invasive devices, severity of pathologies, and prolonged lengths of hospital stays, all of which are well-documented factors that amplify microbial transmission [22]. Neurosurgery departments present an intermediate level of risk, associated with both invasive interventions and greater variability in a patient’s length of stay, which generates a constant but significantly lower contribution than ICUs [24]. Neurology departments maintain a low and stable incidence rate, consistent with the predominantly non-invasive profile of care. This stratification suggests that infection-control interventions should be calibrated differentially, with prioritization of intensive measures in ICUs and targeted approaches in neurosurgery, whereas routine monitoring and prevention strategies are appropriate in neurology [34].

Based on the analysis of hospitalization days, the parallel increase in the total length of hospital stays and the interval to infection detection indicates a structural change in patient profiles and in the dynamics of HAIs. The linear upward trends, with high-value coefficients of determination, suggest that these developments are not random; they reflect a stable trend of increasing case complexity, especially in critically ill departments. The longer duration of hospital stays amplifies the risk of exposure to predisposing factors, invasive devices, prolonged antibiotic therapy, and immobilization, which may contribute to an increase in the proportion of CR/MDR strains observed during the same period. In parallel, the longer intervals to infection detection may reflect both the severity of the underlying pathologies and the difficulty of early identification of infections in patients with a complex clinical course. This convergence between the length of hospital stays, delayed detection, and increasing antimicrobial resistance underlines the need for integrated surveillance strategies, optimization of clinical flows, and antibiotic stewardship interventions, especially in high-risk areas.

There are particularities in regard to neurosurgery. In neurosurgery departments, the prolonged length of hospital stays and the complexity of interventions contribute to a distinct risk profile for HAIs. Neurosurgical patients frequently present with prolonged immobilization, the need for the use of invasive devices, and an increased susceptibility to postoperative complications, all of which are factors that may prolong the interval to infection detection. The increase in the total length of hospitalization has a direct impact on the probability of colonization and infection, and careful monitoring of clinical evolution, together with strict aseptic and device management protocols, becomes essential to reduce the infectious risk.

The days for detection/discharge and hospitalization days suggest two distinct patterns of onset: an early one, associated with critically ill patients or with pre-existing colonization; and a late one, characteristic of prolonged hospitalizations and cumulative exposure to risk factors [35]. The high proportion of cases detected in the first 7 days (38.0%) may reflect either the severity of the underlying pathologies or an increased susceptibility to infections immediately after admission, especially in wards with unstable patients. In contrast, the significant percentage of cases detected after more than 30 days (33.7%) indicates a persistent infectious risk in patients with complex evolution, prolonged immobilization, or the need for invasive devices. The intermediate intervals (8–29 days) account for approximately one third of cases and suggest a transition between early and late infections, reflecting both the dynamics of medical procedures and the variability of clinical severity. This bimodal distribution is consistent with the literature on HAIs in critical care units, where both rapid and late onset requires differentiated prevention and surveillance strategies [35].

In neurosurgery, the distribution by intervals is influenced by the specifics of patients with severe neurological pathology, who frequently present with prolonged immobilization, the need for mechanical ventilation, and the use of invasive devices. These characteristics favor both early infections, through immediate postoperative risks, and late infections, through cumulative exposure to risk factors. Intervals > 30 days are particularly relevant in this context, as they reflect the prolonged and complex course of neurosurgical patients, in whom the infectious risk increases proportionally with the length of hospitalization and the need for intensive care.

The distribution of clinical forms of HAIs with *P. aeruginosa* highlights an etiological profile dominated by respiratory pathology, especially VAP and LRTI, which together represent over two thirds of all cases. This predominance is consistent with the literature, with *P. aeruginosa* being a typical pathogen for infections associated with mechanical ventilation and for patients with severe neurological impairment, where aspiration mechanisms, oropharyngeal colonization, and the prolonged use of respiratory devices significantly increase the risk of HAIs. Urinary and surgical site infections occur less frequently but remain relevant in the context of patients with comorbidities and neurosurgical interventions. The presence of post-neurosurgical meningitis and pressure ulcer-associated infections underlines the vulnerability of patients with severe neurological deficit and prolonged immobilization. This heterogeneous distribution indicates the need for differentiated prevention strategies, with an emphasis on preventing VAP in the ICU, optimizing the management of urinary devices and strengthening asepsis measures in neurosurgery.

In terms of antibiotic resistance, the distribution of the resistance profiles of *P. aeruginosa* isolates over the studied five years highlights a complex evolution, with significant changes in the structure of circulating phenotypes. The progressive increase in CR/MDR strains, visible in both the tables and graphs of the manuscript, indicates a sustained antimicrobial pressure in the hospital environment, especially in critical care units [36,37,38,39,40]. The abrupt rise in this phenotype in 2024 suggests either a change in the local ecology of the strains or the accumulation of favorable factors, such as the prolonged use of carbapenems, exposure to combination therapies, or the persistence of clones adapted to the intensive care environment. Carbapenem-only resistant (CR) strains show a different trend, with marked annual variations and a moderate upward trend, reflected in the regression line in the graph. This evolution may indicate an ecological competition between CR and CR/MDR phenotypes, in which strains with extended-spectrum resistance become progressively dominant over certain intervals, especially in clinical settings with intense exposure to second- and third-line antibiotics. The low presence of ESBL + CR phenotypes suggests that resistance mechanisms mediated by extended-spectrum β-lactamases are not a major determinant in this hospital, and that their emergence in recent years indicates the need for continued monitoring [41].

The high proportion of isolates without resistance data is an important limitation, as it may mask emerging trends or underestimate the true prevalence of MDR phenotypes. However, their relatively constant distribution suggests that the absence of data does not significantly influence the overall direction of the observed trends.

Our results show that the antibiotic resistance of *P. aeruginosa*-positive isolates in hospitals is dynamic, with a visible increase in CR/MDR isolates in recent years, which requires the strengthening of antibiotic stewardship strategies, optimization of the use of carbapenems, and intensification of infection-control measures in high-risk wards, especially in intensive care. This evolution is similar with trends reported at the European level, where *P. aeruginosa* remains one of the main agents involved in severe infections, with a high potential for the development and dissemination of resistance.

The limitations of our study are mainly related to its retrospective design, which does not allow for the uniform control of all clinical and epidemiological variables and depends on the accuracy of data recorded in real-time in clinical and microbiological registries. The distribution of HAIs may reflect the particularities of a tertiary center that admits high-risk patients transferred from multiple health units. This population structure may influence both the detection intervals and the proportion of late infections, limiting direct comparability with hospitals with different profiles or with populations with a lower severity. Variations in addressability and patient flows during the analyzed period may introduce subtle differences in the annual dynamics of cases. Therefore, the observed associations and temporal patterns should be interpreted as descriptive and exploratory and should not be considered evidence of causal relationships.

The possibility of distinguishing colonization from true infection, particularly for isolates obtained from non-sterile sites, represents an additional limitation of this retrospective design. We consider that our study also has a similar limitation, as the identification of *P. aeruginosa* at non-sterile sites does not always indicate the presence of a HAI, and may therefore have contributed to potential overdiagnosis.

Specific limitations of our study may relate to the impact of the COVID-19 pandemic on addressability, the prioritization of admissions, and the availability of resources. The high proportion of patients coming from neurosurgery departments introduces particularities related to the severity of pathologies, prolonged immobilization, and frequent use of invasive devices, which are elements that may increase the probability of late HAIs and could modify the distribution of intervals over 30 days. These characteristics reflect the specifics of a performing tertiary center, but they limit the direct extrapolation of the results to units with a different profile.

## 5. Conclusions

The demographic profile highlighted the predominance of cases in men and a relatively balanced distribution by residence environment, with a punctual inversion in 2024. The high proportion of neurosurgical patients and the context of major cranial interventions may explain the frequency of late infections and the occurrence of cases of postoperative meningitis. Antimicrobial-resistant phenotypes were observed among *P. aeruginosa* isolates, highlighting the need for continued surveillance of antimicrobial resistance and infection-control measures. In addition to continued surveillance, these results further highlight the need for tailored prevention strategies in wards with critically ill neurosurgical patients and long-term hospitalizations.

Our study is distinguished by providing the first integrated epidemiological characterization of HAIs with *P. aeruginosa* in a regional neurosurgical hospital, combining interval analysis, demographic distribution, and the antimicrobial resistance context, making it a benchmark for future evaluations.

## Figures and Tables

**Table 1:** Statistical data on the number of patients and the number of HAI cases, including *Pseudomonas*-positive isolates in the period of 2020–2024.

Years	No. of Patients	HAI Cases		HAIs with *P. aeruginosa*		
		No.	%	No.	%	Incidence * %
2020	5786	255	4.41	12	4.71	0.207
2021	6632	347	5.23	24	6.91	0.362
2022	8467	375	4.43	35	9.33	0.413
2023	10,343	342	3.31	42	12.28	0.406
2024	11,623	334	2.37	50	14.97	0.430
**Total**	**42,851**	**1653**	**3.85**	**163**	**9.86**	**0.380**

* *p* = 0.0038.

**Table 2:** Distribution of cases by age groups of patients included in the study during the period of 2020–2024.

Year/Age Group	2020	2021	2022	2023	2024	Total
0–19	0	0	1	1	0	2
20–44	2	3	3	3	11	22
45–64	5	12	26	18	19	80
65–84	5	9	19	19	19	71
85+	0	0	1	1	1	3
Total	12	24	35	42	50	163

**Table 3:** Number of days from detection to discharge compared to total number of days of hospitalization for HAI cases with *P. aeruginosa.*

Years	Days for Detection/Discharge	Total Days of Hospitalization
2020	186	531
2021	319	824
2022	475	1073
2023	482	1457
2024	867	2018
**Total**	**2329**	**5903**

## Data Availability

All data are available from the first authors and the corresponding authors.

## Abbreviations

The following abbreviations are used in this manuscript:

HAI	Healthcare-associated infection
CR	Carbapenem-resistant
MDR	Multidrug-resistant
ESBL	Extended-spectrum beta-lactamase
ICU	Intensive care unit
N/A	Not available
R^2^	R squared; coefficient of determination
x	Independent variable
y	Dependent variable
